# Obstructive sleep apnea in a mouse model is associated with tissue-specific transcriptomic changes in circadian rhythmicity and mean 24-hour gene expression

**DOI:** 10.1371/journal.pbio.3002139

**Published:** 2023-05-30

**Authors:** Bala S. C. Koritala, Yin Yeng Lee, Laetitia S. Gaspar, Shweta S. Bhadri, Wen Su, Gang Wu, Lauren J. Francey, Marc D. Ruben, Ming C. Gong, John B. Hogenesch, David F. Smith

**Affiliations:** 1 Division of Pediatric Otolaryngology-Head and Neck Surgery, Cincinnati Children’s Hospital Medical Center, Cincinnati, Ohio, United States of America; 2 Department of Otolaryngology-Head and Neck Surgery, University of Cincinnati College of Medicine, Cincinnati, Ohio, United States of America; 3 Division of Human Genetics, Cincinnati Children’s Hospital Medical Center, Cincinnati, Ohio, United States of America; 4 Department of Pharmacology and Systems Physiology, University of Cincinnati College of Medicine, Cincinnati, Ohio, United States of America; 5 Center for Neuroscience and Cell Biology, University of Coimbra, Coimbra, Portugal; 6 Department of Pharmacology and Nutritional Sciences, University of Kentucky, Lexington, Kentucky, United States of America; 7 Department of Physiology, University of Kentucky, Lexington, Kentucky, United States of America; 8 Division of Pulmonary Medicine, Cincinnati Children’s Hospital Medical Center, Cincinnati, Ohio, United States of America; 9 The Sleep Center, Cincinnati Children’s Hospital Medical Center, Cincinnati, Ohio, United States of America; 10 The Center for Circadian Medicine, Cincinnati Children’s Hospital Medical Center, Cincinnati, Ohio, United States of America; National Institutes of Health, UNITED STATES

## Abstract

Intermittent hypoxia (IH) is a major clinical feature of obstructive sleep apnea (OSA). The mechanisms that become dysregulated after periods of exposure to IH are unclear, particularly in the early stages of disease. The circadian clock governs a wide array of biological functions and is intimately associated with stabilization of hypoxia-inducible factors (HIFs) under hypoxic conditions. In patients, IH occurs during the sleep phase of the 24-hour sleep–wake cycle, potentially affecting their circadian rhythms. Alterations in the circadian clock have the potential to accelerate pathological processes, including other comorbid conditions that can be associated with chronic, untreated OSA. We hypothesized that changes in the circadian clock would manifest differently in those organs and systems known to be impacted by OSA. Using an IH model to represent OSA, we evaluated circadian rhythmicity and mean 24-hour expression of the transcriptome in 6 different mouse tissues, including the liver, lung, kidney, muscle, heart, and cerebellum, after a 7-day exposure to IH. We found that transcriptomic changes within cardiopulmonary tissues were more affected by IH than other tissues. Also, IH exposure resulted in an overall increase in core body temperature. Our findings demonstrate a relationship between early exposure to IH and changes in specific physiological outcomes. This study provides insight into the early pathophysiological mechanisms associated with IH.

## Abstract 2

Intermittent hypoxia is a major clinical feature of obstructive sleep apnea (OSA). This study uses a mouse model of OSA to unveil time- and tissue-specific changes in the transcriptome, revealing the early biological changes linked to this disorder across multiple organ systems.

## Introduction

Over a billion people worldwide suffer from obstructive sleep apnea (OSA) and its associated health conditions. It is estimated that OSA costs the United States healthcare system $150 billion each year [1,2]. OSA is characterized by episodes of upper airway obstruction during sleep, resulting in intermittent hypoxia (IH) and sleep fragmentation. Patients exposed to IH over long periods may suffer from cardiovascular, respiratory, metabolic, and/or neurologic sequelae [3]. Most importantly, pathophysiological changes associated with chronic, untreated OSA lead to increased rates of morbidity and mortality [4].

In patients with OSA, IH occurs only during the sleep phase of the 24-hour sleep–wake cycle and has the potential to alter physiological circadian rhythms. The circadian clock is a molecular oscillator that uses positive and negative transcriptional–translational feedback loops of the core clock components [5]. The central clock, located in the suprachiasmatic nucleus of the hypothalamus, and peripheral clocks located throughout the body help orchestrate an array of behavioral and physiological rhythms [6]. Circadian clocks are one of the key components in maintaining systemic homeostasis since they act as timekeepers for molecules, cells, organs, and physiological processes [7]. However, the circadian clock can be disrupted by internal factors (e.g., health conditions) and external factors (e.g., living conditions) [8]. Evidence suggests that dysregulation of the circadian clock can increase the risks for heart disease, obesity, cancer, and even dementia [9]. Despite our understanding of the basic mechanisms of the circadian clock, we do not know how dysregulation of the clock contributes to disease in those patients with OSA at risk for comorbid conditions. Hypoxia-inducible factors (HIFs) influence the expression of canonical clock genes [10]. Consequently, the function of the circadian clock can be affected by exposure to IH [11–13]. Several independent studies have evaluated rhythmic changes in known circadian clock genes after exposure to IH in animals [11,12]. However, these studies have not evaluated changes at the genome-wide scale across both anatomic location and time, and the evaluation is limited to a small set of core clock genes.

OSA manifests only during sleep, yet the negative physiological effects persist throughout the 24-hour cycle. To better understand the effects of IH, we must evaluate those molecular changes that occur across the 24-hour day. In nature, the human body is generally exposed to approximately 21% oxygen, but physiological needs for oxygen and resistance to periods of hypoxia are highly tissue specific. For example, lung and liver are more oxygenated compared to the muscle [14]. Since chronic, untreated OSA is primarily associated with negative cardiopulmonary outcomes, we hypothesized that IH would impact the biological processes of lung and heart more rapidly than other tissues. In addition, these early biological changes would likely contribute to the resulting pathophysiological sequelae after chronic exposure to IH. In the current study, we comprehensively evaluated both time- and tissue-specific transcriptomic changes resulting from IH in 6 different mouse tissues, including liver, lung, kidney, muscle, heart, and cerebellum. These transcriptomic changes were used to examine altered biological processes and physiological changes in mammals. The results suggest that the transcriptome of cardiopulmonary tissues and associated biological processes were more impacted in comparison with other evaluated tissues. Our findings provide novel insight into the pathophysiological mechanisms that could be associated with end-organ damage in patients with chronic exposure to IH.

## Results

### Short-term exposure to IH highly affected 24-hour mean expression of the lung transcriptome and associated biological pathways

**Fig 1:**
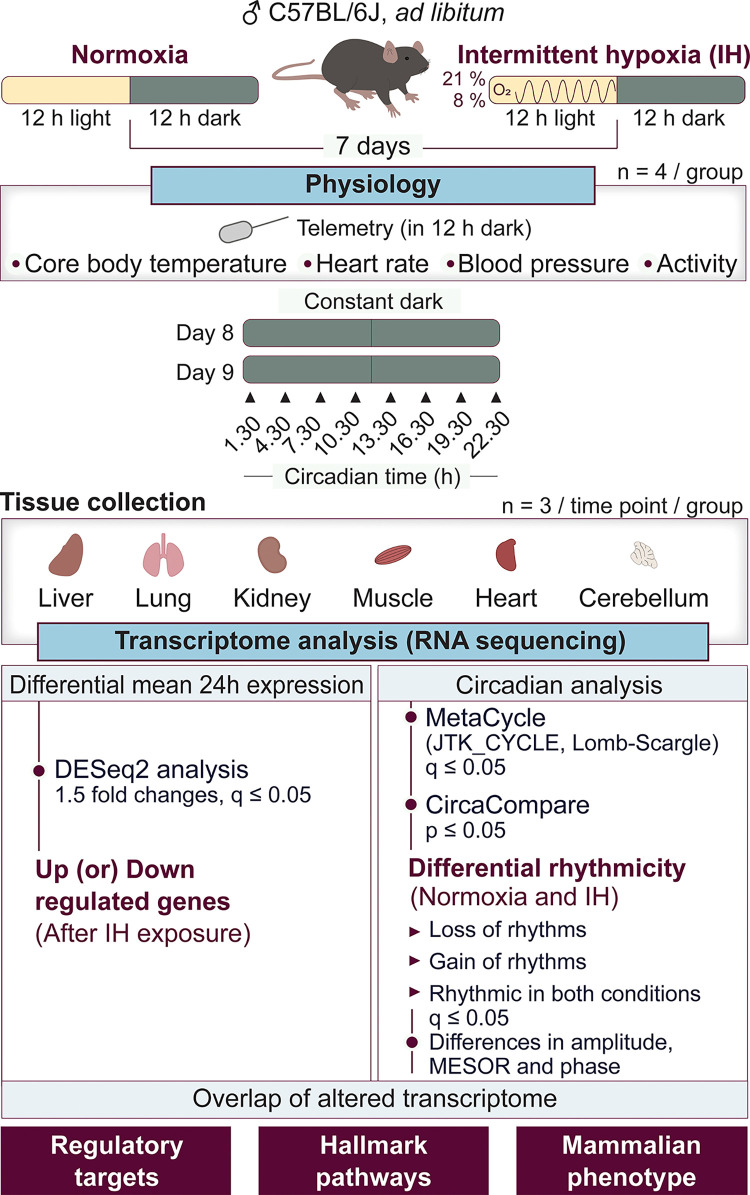
Study design. In this study, we investigated the early effects of IH on physiological parameters (CBT, HR, BP, and activity) and tissue-specific transcriptome in male C57BL/6J mice. Physiological parameters were assessed in mice (*n* = 4 for each condition) after completion of 7 days of normoxia or IH exposure using the HD-X10 DSI telemetry system. We also collected several organs (liver, lung, kidney, muscle, heart, and cerebellum) from mice (*n* = 3 at each time point for each condition) on the second day of constant darkness for 24 hours with 3-hour intervals following exposure to a normoxic or IH condition. RNA was extracted from these samples and sequenced with NovaSeq S4 flow cell for a minimum of 40 million paired-end reads per sample. The data were then evaluated to assess for differences in mean 24-hour expression and circadian rhythmicity between normoxia and IH. Furthermore, we enriched the observed alterations among associated regulatory targets, hallmark pathways, and mammalian phenotypes. BP, blood pressure; CBT, core body temperature; IH, intermittent hypoxia; MESOR, Midline Estimating Statistics of Rhythm; HR, heart rate.

**Fig 2:**
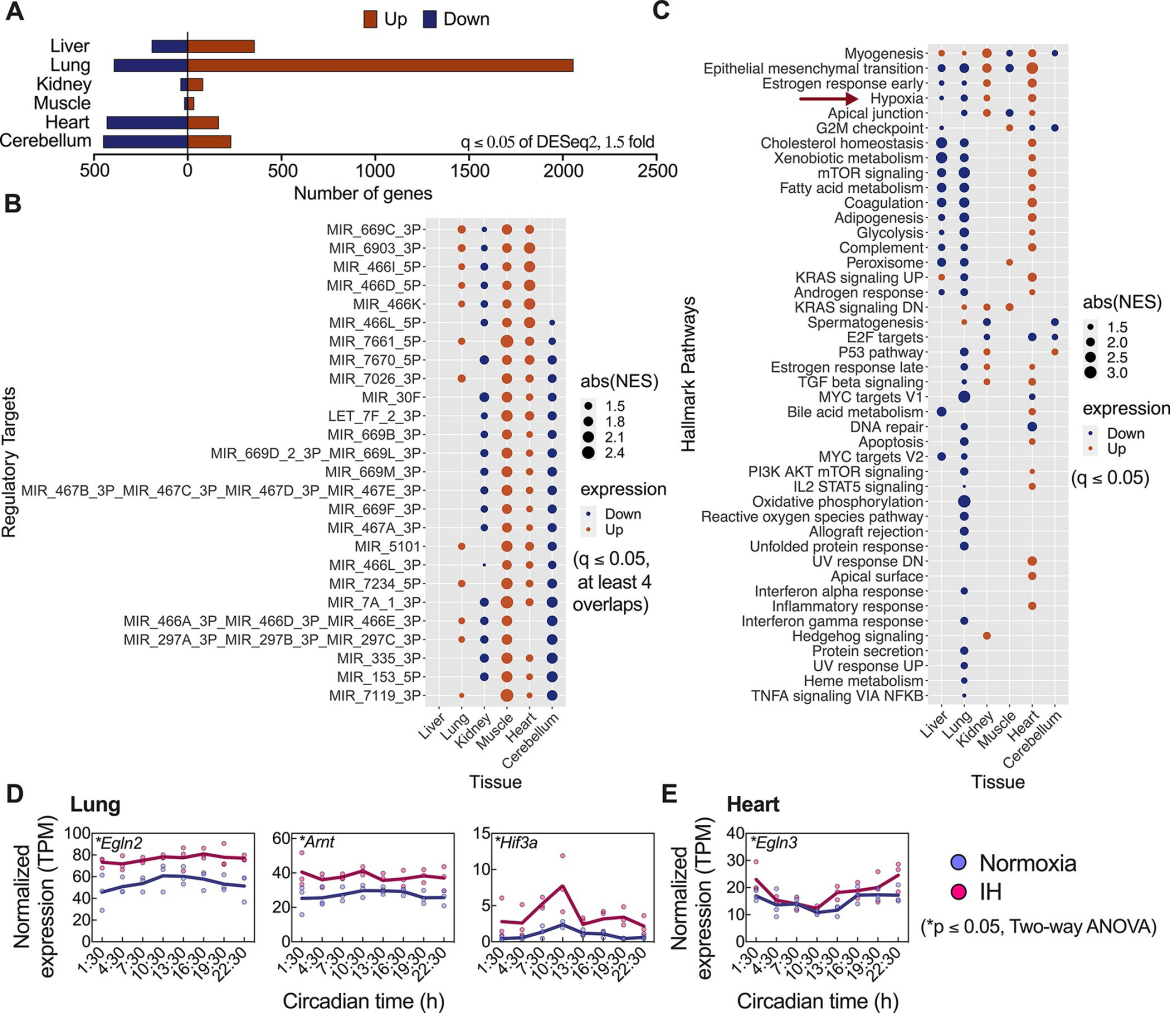
Differential mean 24-hour expression. To determine whether IH impacted mean 24-hour expression, we ran a DESeq2 analysis using the Kallisto R package. **(A)** The transcriptome changes within liver, lung, kidney, muscle, heart, and cerebellum were examined. Based on q ≤ 0.05 and a 1.5-fold difference, significant differences with up- (orange) or down (blue)-regulated genes have been screened and presented. (**B**) GSEA of consistently and significantly (q ≤ 0.05) associated regulatory targets with differential gene expression in at least 4 distinct tissues. The results indicated that in the lung, muscle, and heart, MIRs were primarily associated with up-regulated genes, while the same MIRs were associated with down-regulated genes in the kidney and cerebellum. **(C)** GSEA of hallmark pathways for differentially expressed genes upon IH exposure. Dot size indicates absolute NES. Statistical significance was determined by (q ≤ 0.05, GSEA). Hallmark pathways in the lung and heart are highly impacted compared to those in other organs. Some of these changes in hallmark pathways overlapped with processes in other organs. Significant *(p* ≤ 0.05, two-way ANOVA*)* changes in gene expression of PHDs and/or HIFs in **(D)** the lung and **(E)** the heart after exposure to normoxia and IH. Data output for Fig 2A can be found in S1 Data, for Fig 2B in S2 Data, for Fig 2C in S3 Data. Raw and processed data files for all figures in Fig 2 accessible through GEO series accession number GSE214530. GSEA, gene set enrichment analysis; HIF, hypoxia-inducible factor; IH, intermittent hypoxia; MIR, microRNA; NES, normalized enrichment score; PHD, prolyl hydroxylase.

We investigated the impact of short-term IH on changes in the mean 24-hour expression of whole-genome and associated biological processes in multiple tissues. This approach allowed us to incorporate both circadian and noncircadian changes influenced by IH (**Fig 1**). We conducted differential gene expression analysis on 24-hour time course data from liver, lung, kidney, muscle, heart, and cerebellum of mice exposed to normoxia and IH. A cutoff q ≤ 0.05 of DESeq2 with a 1.5-fold difference was used to determine differentially expressed genes between normoxia and IH. The lung was most affected by IH (15.7% of detected transcripts) compared to liver (4.8%), heart (4.7%), cerebellum (4.6%), kidney (0.9%), and muscle (0.4%). Interestingly, the predominant direction of change (i.e., up- versus down-regulation) varied with tissue type. While IH primarily led to up-regulation in the lung (83.9%), kidney (67.8%), liver (65%), and muscle (64.2%), it primarily led to down-regulation in the heart (72.2%) and cerebellum (66%). Overall, the lung transcriptome was most affected by IH compared to other organs (**Figs 2A and S1 and S1 Data**).

Furthermore, we assessed the effects of short-term IH on transcriptional regulation across multiple tissues using a gene set enrichment analysis (GSEA) of regulatory targets from the GSEA Molecular Signature Database v7.5.1 [15]. Our analysis included both microRNAs (MIRs) and transcription factor targets associated with differential gene expression. We identified a significant (q ≤ 0.05) number of regulatory targets (708 of muscle, 285 of cerebellum, 204 of heart, 171 of kidney, 60 of lung) in all tissues except the liver, which had only 3 targets. Notably, MIRs were found to be the most commonly identified targets, with over 75% of regulatory targets identified across tissues being MIRs, except for the liver (**S2 Data**). We also identified a subset of MIRs that were significantly (q ≤ 0.05) associated with differential expression in at least 4 different tissues, exhibiting tissue-specific transcriptional regulation. Specifically, MIRs in the lung, muscle, and heart were primarily associated with up-regulated genes, while the same MIRs were associated with down-regulated genes in the kidney and cerebellum (**Fig 2B**).

To evaluate the biological state of mice after short-term IH exposure, we conducted GSEA of hallmark pathway gene sets [15]. As a threshold, q ≤ 0.05 was used to identify pathways that were significantly affected by IH exposure. We identified tissue-dependent pathway changes; the most impacted pathways were found in the lung, followed by the heart. Interestingly, 19 of the same GSEA pathways (out of 50) were differentially regulated between the heart and lung after IH exposure, often elevated in one but decreased in the other. We found that the majority of hallmark pathways in the liver, lung, and cerebellum showed enrichment of down-regulated genes, while the pathways in kidney and heart exhibited enrichment of up-regulated genes (**Fig 2C and S3 Data**). Of the 6 pathways, 3 were associated with the enrichment of down-regulated genes in muscle, while the remaining 3 showed enrichment with up-regulated genes. Our study found 6 altered pathways in at least 4 different tissues, including myogenesis, epithelial mesenchymal transition (EMT), estrogen response early (ERE), hypoxia, apical junction, and G2M checkpoints. In the kidney and heart, the pathways of EMT, ERE, and hypoxia were consistently associated with up-regulated genes, whereas the same pathways were associated with down-regulated genes in the liver and lung **(Fig 2C**). These results offer significant understanding of how short-term IH affects transcriptome and pathway regulation in specific tissues, which could aid in the identification of potential targets for health conditions associated with IH.

### Specific prolyl hydroxylases and hypoxia-inducible factors in the lung were highly affected by short-term exposure to IH

Prolyl hydroxylases (PHDs) are well known for sensing molecular oxygen and stabilizing HIFs through hydroxylation [16]. The mechanism of HIF stabilization under sustained hypoxia has been well characterized but has not been fully examined under IH conditions. The expression of HIFs are not constant across different tissues [17], and we hypothesized that the impact of IH on PHDs and HIFs will be different based on the tissue type. We evaluated time and tissue-specific gene expression of PHDs and HIFs in 6 different tissues following IH exposure. Interestingly, there are a greater number of PHDs or HIFs (*Egln2*, *Arnt*, and *Hif3a*) significantly (*p* ≤ 0.05, two-way ANOVA) altered in the lung after IH exposure in comparison with other organs (**Figs 2D and S2**). In addition, *Egln3* from the heart (**Figs 2E and S2**) and *Egln2* from cerebellum (**S2 Fig**) were significantly (*p* ≤ 0.05, two-way ANOVA) altered following IH exposure. Based on these findings, cardiopulmonary tissues, particularly the lung, is highly responsive to IH.

### The circadian clock in the lung is highly impacted by short-term IH exposure

**Fig 3:**
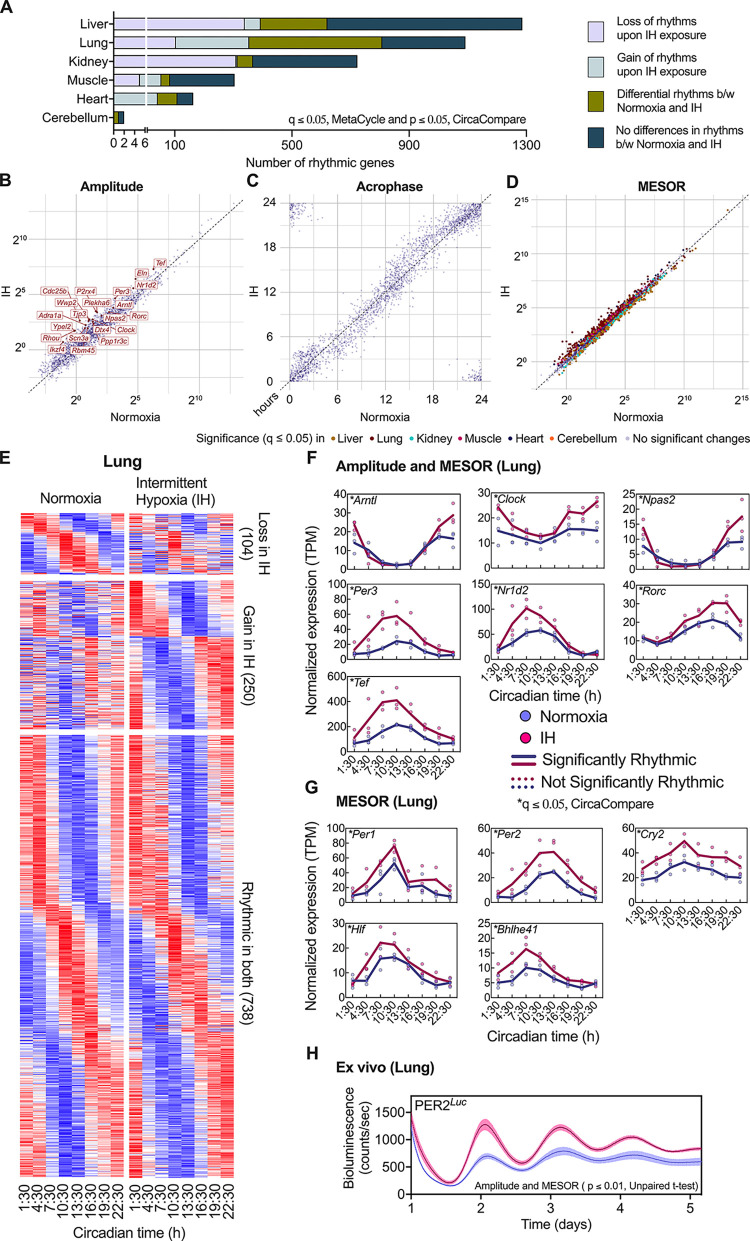
Differential circadian transcriptome. **(A)** Differentially rhythmic genes were identified in the liver, lung, kidney, muscle, heart, and cerebellum after exposure to IH. Genes with loss or gain of rhythms were evaluated by combining MetaCycle (q ≤ 0.05) and CircaCompare (*p* ≤ 0.05). The circadian properties of the differentially rhythmic genes in both conditions were evaluated, including **(B)** amplitude, **(C)** acrophase, and **(D)** MESOR, using a cutoff threshold of q ≤ 0.05 of CircaCompare. Correlation plots were used to illustrate the differences between the 2 conditions. Significant changes in amplitude were observed only in the lung following IH exposure, while significant changes in MESOR were observed in all organs. **(E)** The lung heatmap illustrates those genes with significant (q ≤ 0.05 of MetaCycle and *p* ≤ 0.05 of CircaCompare) loss (top panel) or gain (middle panel) of circadian rhythms following IH exposure, as well as rhythmic genes under normoxic and IH conditions (bottom panel). **(F and G)** Among the canonical clock genes, *Arntl*, *Clock*, *Npas2*, *Per3*, *Nr1d2*, *Rorc*, and *Tef* in the lung exhibited significant changes (q ≤ 0.05, CircaCompare) in amplitude and MESOR following IH exposure. Additionally, *Per1*, *Per2*, *Cry2*, *Hlf*, and *Bhlhe41* showed significant differences in MESOR. **(H)** Bioluminescence of PER2 protein in lung tissue from PER2^*Luc*^ animals exposed to normoxia vs. IH showed significant increases in both amplitude and MESOR (*p* ≤ 0.01, unpaired *t* test) following IH exposure. Data output for Fig 3A-3G can be found in S4 Data. Raw and processed data files for Fig 3A-3G accessible through GEO series accession number GSE214530. Data for Fig 3H can be found in S5 Data. IH, intermittent hypoxia; MESOR, Midline Estimating Statistics of Rhythm; TPM, transcripts per million.

Approximately half of the transcriptome in mammals exhibit circadian rhythms, and several biological and physiological processes are influenced by the circadian clock. We performed RNA sequencing for a 24-hour time course on samples from the liver, lung, kidney, muscle, heart, and cerebellum from mice after exposure to a 7-day normoxic or IH condition. After removing genes with consistently low transcript levels, circadian analyses were conducted on at least 12,000 genes in each tissue. We used an integrative analytic approach to compare circadian rhythms between normoxic and IH conditions. Genes that passed through the q ≤ 0.05 of MetaCycle [18] in at least 1 condition were analyzed for differential rhythms using *p* ≤ 0.05 of CircaCompare [19]. We examined the effects of IH exposure on rhythmic patterns, encompassing both loss and gain of rhythms, as well as analyzing variations in rhythmic parameters such as acrophase, amplitude, and Midline Estimating Statistics of Rhythm (MESOR) between the rhythmic genes under both conditions (**Figs 3 and S3**).

IH had the greatest impact on the circadian transcriptome of the lung. Specifically, IH affected approximately 74% of the rhythmic genes in the lung, 66.9% in the heart, 50.8% in the kidney, 48.3% in the liver, and 27.6% in the muscle. IH resulted in a significant loss (q ≤ 0.05 of MetaCycle and *p* ≤ 0.05 of CircaCompare) of rhythmic genes in the kidney (42.9%) and liver (26.4%) but a significant gain (q ≤ 0.05 of MetaCycle and *p* ≤ 0.05 of CircaCompare) of rhythms in previously unrhythmic genes in the heart (25.8%), lung (22.9%), and muscle (16.1%). Only 2 rhythmic genes were detected in the cerebellum, but none of them lost or gained rhythmicity following IH exposure. Despite the large-scale tissue-specific transcriptomic changes, there were still many genes that were detected as rhythmic in both control and IH conditions (**Figs 3A, 3E, and S3A–S3E and S4 Data**). Some of these genes showed significant (q ≤ 0.05, CircaCompare) differences in amplitude, acrophase, or MESOR. However, among all organs, the lung exhibited the greatest variability in circadian rhythms (**Fig 3A–3D**). Interestingly, no amplitude differences were detected in any tissues except the lung (**Fig 3B**). We used a stringent criterion of “at least 4 hours” for evaluating acrophase difference between normoxia and IH. There were no discernible acrophase differences in response to IH, including among core clock genes (**Fig 3C**). On the other hand, a substantial portion of rhythmic genes from each tissue showed differences in MESOR (**Fig 3D**). These results suggest that even short-term IH exposure profoundly altered the circadian transcriptome of cardiopulmonary tissues that are susceptible to OSA comorbidities.

We further evaluated the effects of IH on tissue-specific molecular clocks by examining 17 canonical clock genes (*Arntl*, *Clock*, *Per1*, *Per2*, *Per3*, *Cry1*, *Cry2*, *Nr1d1*, *Nr1d2*, *Dbp*, *Npas2*, *Rorc*, *Tef*, *Hlf*, *Nfil3*, *Bhlhe41*, and *Ciart*) that are known to be involved in regulating the circadian clock in mammals. At least 11 of these 17 clock genes were significantly rhythmic (q ≤ 0.05 of MetaCycle and *p* ≤ 0.05 of CircaCompare) in every organ except the cerebellum. As expected, IH had the greatest impact on the clock in the lung, the primary location of hypoxic insult, followed by the clock in the heart. In the lung, IH affected the amplitude and MESOR of *Arntl*, *Clock*, *Npas2*, *Per3*, *Nr1d2*, *Rorc*, and *Tef* (**Fig 3F**), while *Per1*, *Per2*, *Cry2*, *Hlf*, and *Bhlhe41* only showed MESOR differences (**Fig 3G**). We validated the impact of IH on the circadian rhythm of lung PER2 at the protein level. PER2^*LUC*^ mice were exposed to short-term normoxic or IH conditions followed by a real-time luciferase recording for up to 5 days from lung explants. The results suggested a significant (*p* ≤ 0.01, unpaired *t* test) increase in amplitude and MESOR of PER2 rhythms in the days following IH exposure (**Fig 3H, S5 Data**). In the heart, *Cry1* and *Rorc* demonstrated gain of rhythms (**S3F Fig**), while *Cry2*, *Hlf*, *Bhlhe41*, and *Nfil3*, exhibited differences in MESOR after IH exposure (**S3G Fig**). Furthermore, we also identified a MESOR difference of only *Hlf* in the muscle (**S3H Fig**). No significant differences in amplitude, acrophase, or MESOR were identified for canonical clock genes in the liver, kidney, or cerebellum. The results suggest that multiple targets among the molecular clock demonstrated changes in amplitude and/or MESOR in the lung and heart compared to other peripheral tissues, offering insights on why the circadian transcriptome of these organs are more affected by IH.

### Lung tissue demonstrated a robust correlation between genes with differential rhythms and mean 24-hour expression following IH exposure

**Fig 4:**
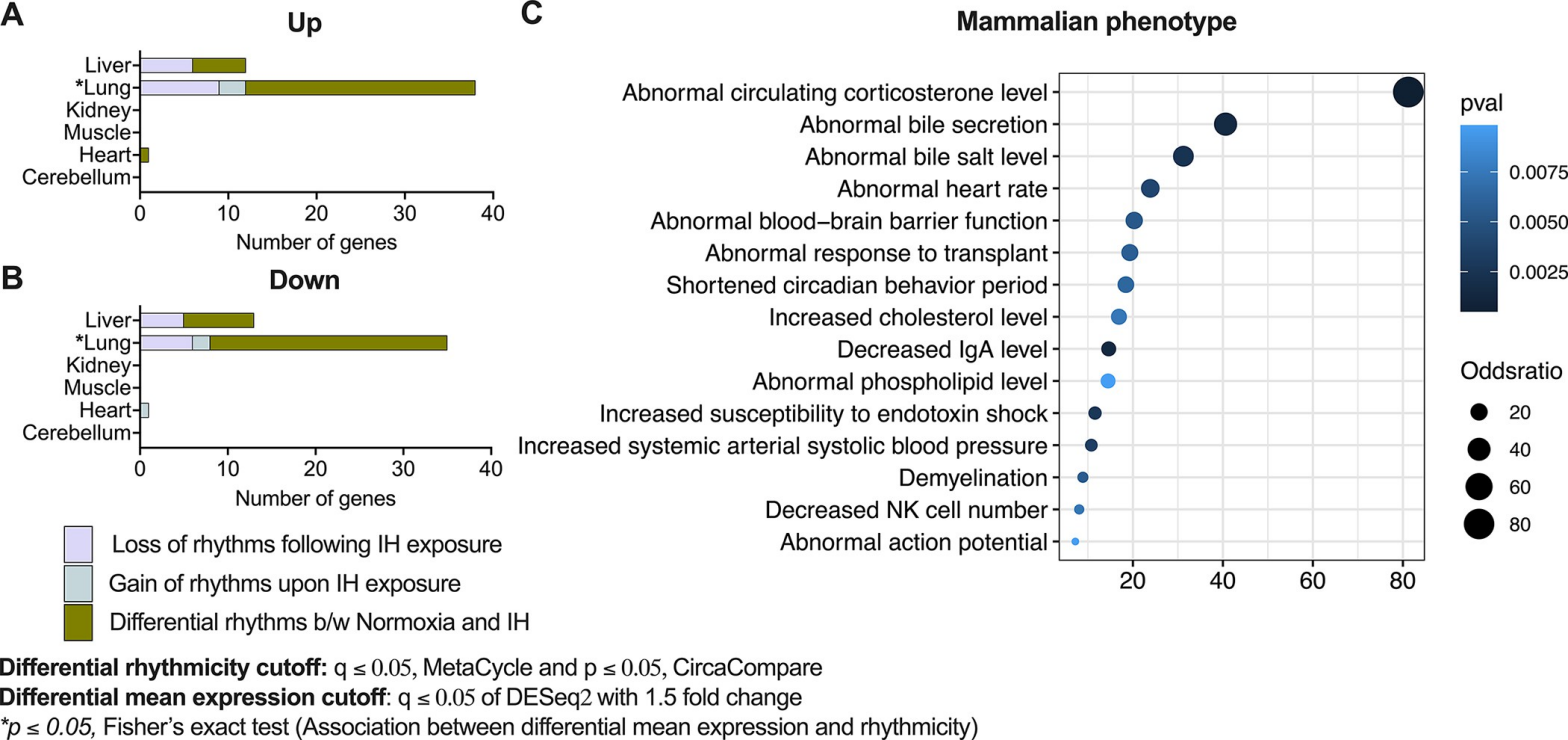
Genes with differences in rhythms and mean 24-hour expression. IH exposure resulted in significant changes in rhythmicity (q ≤ 0.05 of MetaCycle and *p* ≤ 0.05 of CircaCompare for presence of rhythmicity; q ≤ 0.05 of CircaCompare for differential circadian properties) and mean 24-hour expression (q ≤ 0.05 with 1.5-fold difference of DESeq2) of only a few genes in the liver, lung, and heart but not in the kidney, muscle, or cerebellum. In comparison with other organs, the lung demonstrated a higher number of altered genes and a significant (*p* ≤ 0.05, Fisher’s exact test) correlation between circadian rhythmicity and mean 24-hour expression. **(A)** Up-regulated genes with differential rhythms. **(B)** Down-regulated genes with differential rhythms. **(C)** Enrichment of genes to the mammalian phenotype with differential rhythms and changes in mean 24-hour expression across the tissues (*p* ≤ 0.01). Data output for Fig 4A and 4B associated with S1 Data and S4 Data. Data output associated with Fig 4C can be found in S6 Data. Raw and processed data files associated with Fig 4 accessible through GEO series accession number GSE214530. IgA, immunoglobulin A; IH, intermittent hypoxia; NK, natural killer.

To determine the relationship between differential rhythms and mean 24-hour expression, a Fisher’s exact test was performed for each tissue separately. The findings indicated a significant correlation (*p* ≤ 0.05, Fisher’s exact test) between genes with distinct rhythmic patterns and those with varied mean 24-hour expression, but only in the lung, not in other tissues. Additionally, we investigated for gene sets that differed in both mean 24-hour expression and circadian rhythmicity across the tissues. We discovered overlapping gene sets in the lung, liver, and heart following IH exposure, but not in the kidney, muscle, or cerebellum. The lung had a greater number of differentially regulated genes compared to other organs (**Figs 4A, 4B, and S4–S6**). Interestingly, we found that only a few clock genes (*Per2*, *Per3*, and *Tef*) and 1 HIF (*Hif3a*) were significantly (q ≤ 0.05 of DESeq2 with 1.5-fold) up-regulated in the lung, demonstrating significant differences (q ≤ 0.05, CircaCompare) in amplitude and/or MESOR (**S4C and S4D Fig**). These genes could serve as potential molecular markers for evaluating IH-specific changes in health conditions.

We further enriched for gene sets that differ in mean 24-hour expression and circadian rhythmicity in the lung, liver, and heart to the mammalian phenotype using Enrichr [20]. A *p* ≤ 0.01 was used to determine the possible altered phenotypes in mammals. Our analysis revealed that specific genes that have altered 24-hour mean expression and circadian rhythms in the liver, lung, and heart were enriched with mammalian phenotypes (**Fig 4C, S6 Data**). Notably, some of these enriched pathways include abnormal heart rate (HR), abnormal blood pressure (BP), abnormal circadian phase of behavior (circadian phase of activity and sleep), and abnormal stress hormone levels (**Fig 4C**). These phenotypes are often observed in patients with OSA that exhibit associated comorbid conditions.

### Core body temperature is increased upon short-term exposure to IH

**Fig 5:**
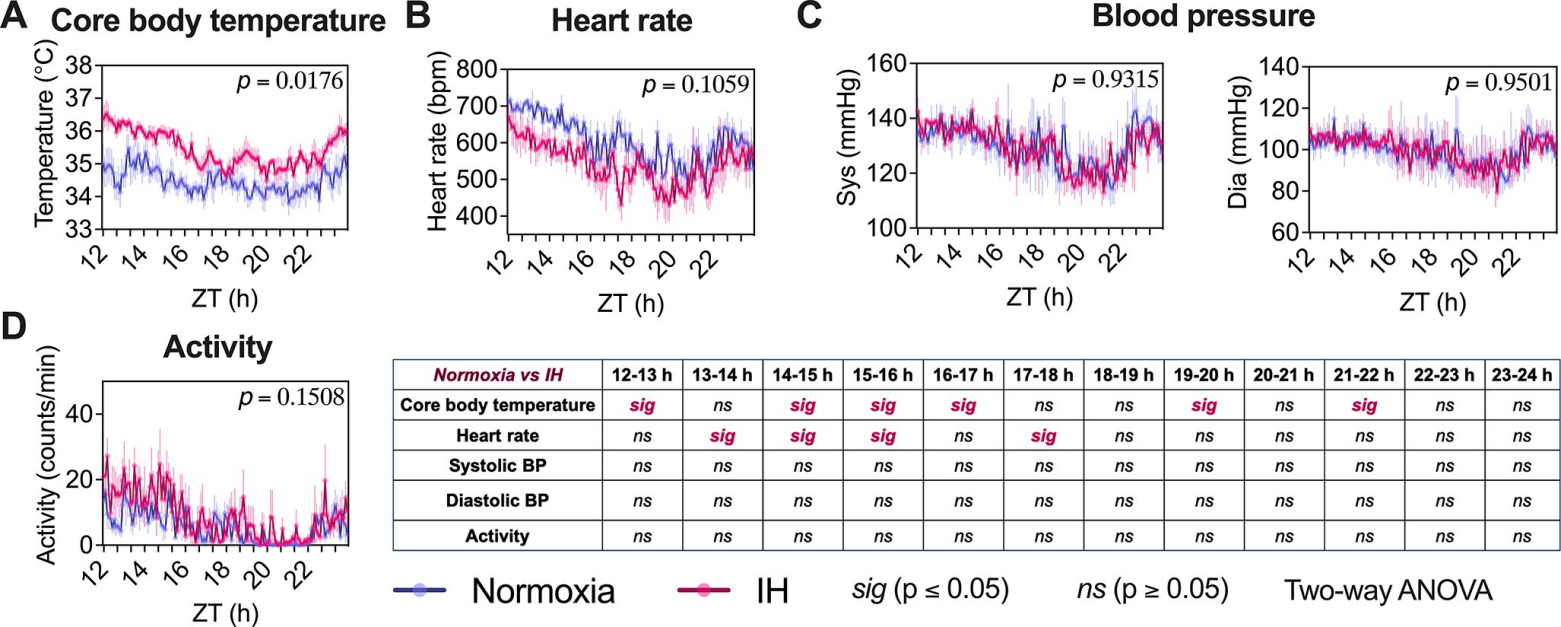
Physiological parameters. Physiological properties of mice (*n* = 4 for each condition) were assessed on the seventh day of their active phase (ZT12 to 22 hours) using an HD-X10 DSI telemetry system. We measured **(A)** CBT, **(B)** HR, **(C)** systolic and diastolic BP, and **(D)** activity using an average 1-hour bin analysis after exposing the mice to IH. Although systolic and diastolic BP and activity did not show any significant differences in any time bins between normoxia and IH, CBT and HR did show significant differences (*p* ≤ 0.05, two-way ANOVA) across several time bins. In addition, we also observed an overall significant increase (*p* ≤ 0.05, two-way ANOVA) in CBT following IH exposure. Data for Fig 5A–5D can be found in S7 Data. BP, blood pressure; CBT, core body temperature; HR, heart rate; IH, intermittent hypoxia; ZT, Zeitgeber time.

OSA is strongly associated with BP dysregulation in children and hypertension and stroke in adult patients with untreated disease [21,22]. Animal models of IH have also shown an association between IH and similar adverse cardiopulmonary outcomes. However, we do not understand the course of these longitudinal physiological changes that occur prior to end-organ damage. After a 7-day exposure to IH, we measured core body temperature (CBT), HR, systolic and diastolic BP, and activity during the active phase of mice using a DSI HD-X10 telemetry probe (**Fig 5A–5D, S7 Data**) as described in our **Methods**. While the overall animal activity, HR, and systolic and diastolic BP were unaffected by a 7-day exposure to IH, our 1-hour bin analysis revealed a persistent and significant decrease in HR for 3 consecutive bins between the 13th to 16th hours of inactivity (*p* ≤ 0.05, two-way ANOVA) following IH exposure (**Fig 5B–5D**). Additionally, we observed a significant increase (*p* ≤ 0.05, two-way ANOVA) in CBT in response to the 7 days of IH exposure, both overall and for 3 consecutive bins between the 14th to 17th hours (**Fig 5A**). Strikingly, the overall change of body temperature was consistent with observations of increased body temperature in patients with OSA [23–25]. Body temperature and lung ventilation are intimately related through interactions with sleep–wake state, metabolism, and thermoregulation. In addition, body temperature is a key regulator of several other biochemical and biological processes, including peripheral oscillators of the circadian clock [26].

## Discussion

A key challenge to preventive care in patients with OSA is “early diagnosis” prior to the development of associated medical comorbidities. Although children with OSA may not develop cardiovascular disease, they can exhibit changes in several risk factors such as BP variability and decreased nocturnal BP dipping [27]. These risk factors may further contribute to more serious diseases, such as hypertension, metabolic syndrome, and other cardiovascular dysfunction in adulthood [28,29]. However, the early biological changes that lead to disease processes are not yet fully understood, and assessing tissue-specific mechanisms that contribute to OSA-associated disease pathology is extremely difficult. In this study, we investigated the impact of short-term exposure to IH on the whole-genome transcriptome and associated hallmark pathways in an established animal model of OSA. These data provide insights into the early changes in biological pathways that could be used to identify targets for future mechanistic studies evaluating diagnostic or therapeutic approaches. Our findings suggest that there is time- and tissue-specific variability of the whole-genome transcriptome in multiple organ systems following even a short exposure to IH, with the lung transcriptome being the most affected in comparison with other organs. We also measured physiological changes associated with OSA; interestingly, we observed an overall increase in CBT following IH exposure as seen in some patients with OSA [23–25].

There are several animal models available for studying OSA-associated pathophysiological outcomes, with each model having its own strengths and weaknesses. One alternative model involved the use of implanted tracheal balloons [30], while the most commonly used model exposes animals to episodes of hypoxia followed by recovery to room air [31]. Both of these models can result in disturbances in vascular and metabolic function, such as changes in BP, sympathetic dysregulation, and metabolic disturbances [32–36]. IH models eliminate the need for surgical implantation or airway scarring, allowing for the investigation of oxygen desaturation, particularly with respect to HIF-associated pathways as well as the circadian system.

IH is a major clinical feature of OSA. The presence of HIFs under hypoxic conditions can impact the circadian system through regulation of the canonical clock genes [10] and inflammatory responses [37]. Changes in oxygen levels can serve to synchronize circadian rhythms in an HIF-dependent manner [38]. However, more significant episodic variations in oxygen levels frequently associated with OSA can have adverse effects on peripheral tissues due to their physiological requirements [14], leading to variability in hypoxic responses and resistance to hypoxia-induced damage. Additionally, sustained hypoxia and IH can affect HIF-dependent pathways differently. Previous research has not explored the tissue-specific responses of oxygen molecule sensors, PHDs and HIFs, in response to IH. In the current study, we found that the expression of these molecules is highly variable across tissues following IH exposure. Given the strong association between HIFs and the circadian clock [10], changes in HIF levels could contribute to the variability in the circadian clock regulation following exposure to IH.

OSA manifests only during sleep, but its negative impact on physiology persists across the 24-hour day. A recent study has shown that even an acute, one-time, 4-hour exposure to IH alters the circadian rhythms of some canonical clock genes in the lung, liver, and kidney [12]. Though assessing acute responses are important, patients with OSA experience IH throughout their daily sleep cycles. We examined the changes in mice in a well-established model that replicates features of OSA [11,13,39–44]. However, none of the previous studies using this model have demonstrated downstream effects of altered clock genes following IH exposure, which could provide significant insight into the disease progression. Given that approximately 50% of the mammalian transcriptome is rhythmic [45], we conducted a time-course study and evaluated IH-specific time-dependent and time-independent changes of whole-genome transcriptome across multiple organ systems. We discovered that a 7-day exposure to IH is sufficient to trigger significant transcriptomic changes in a tissue-specific and time-dependent manner. Even with just 7 days of exposure, the lung transcriptome was the most affected compared to other tissues.

Changes in the circadian system are often characterized by differences in phase, but the importance of amplitude as an indicator of oscillation robustness has been understudied. Alterations in amplitude relative to period length defines the loss or gain of rhythmicity, and only a few studies have explored the relationship between changes in circadian amplitude and physiological disease state [46]. In this study, we investigated the impact of IH exposure on the rhythmic transcriptome by measuring both the loss and gain of rhythmicity and the differences in circadian properties, such as acrophase, amplitude, and MESOR. Although acute IH exposure has shown phase differences in canonical clock genes [12], our high-temporal resolution data suggests tissue-specific differential rhythmicity with no discernible acrophase differences, including among the clock genes. However, there are clear differences in amplitude and/or MESOR among all tissues, particularly in the lung, indicating that the strength of the circadian system is affected even at an early stage of IH exposure in cardiopulmonary tissues. Changes in physiological rhythms over time, such as the loss of nighttime dipping of BP (nondipping) [27], in patients with OSA can increase the risks of cardiovascular disease or acute cardiac events [47].

In addition, the development of disease pathology can also be influenced by several noncircadian factors. To explore their potential role, we analyzed how IH affects the 24-hour mean expression of the whole transcriptome across multiple organ systems. We then linked these alterations to regulatory targets and hallmark pathways. Interestingly, more than 75% of the regulatory targets were identified as MIRs in at least 5 distinct organ systems. This finding is particularly compelling because MIRs are considered potential biomarkers for comorbidities associated with OSA, especially cardiovascular diseases [48–51]. Interestingly, myogenesis, EMT, ERE, and hypoxia pathways were dysregulated in our tissues of interest (cardiopulmonary system, liver, kidney, and/or muscle). Previous studies suggest that dysregulation of these hallmark pathways contributes to the development of cardiovascular disease, diabetes, and kidney disease [52–55]. Nevertheless, more functional validation studies are necessary to evaluate these pathways and assess IH-related health conditions. Besides studying these hallmark pathways, we also evaluated potential phenotypic changes that are often seen later in the disease process. We found that early transcriptome changes contribute to phenotypes associated with OSA, such as abnormal HR and BP. Our study identified pathways that change prior to the development of other end-organ damage.

Rodent studies involving IH have predominantly evaluated BP using tail-cuff measurements [56,57]. However, this technique has limitations in terms of reliability and reproducibility [58]. We used an implantable telemetry system [59] to continuously monitor CBT, BP, HR, and activity in response to short-term exposure to IH. Based on our transcriptomic data and changes seen in biological processes, these phenotypes could be attributed to early molecular changes that occur as a consequence of IH exposure. Although we did not observe significant changes in the overall effect of systolic and diastolic BP, HR, or activity after a 7-day exposure to IH, we hypothesize that such changes may take longer to manifest and require consistent exposure over an extended period of time, as seen in other chronic IH studies [56,60]. Interestingly, we did observe a significant increase in CBT after IH exposure, after only 7 days. This finding is consistent with physiological responses observed in patients with OSA [23–25]. CBT plays a critical role in maintaining biological homeostasis [61] and can influence the expression of genes, proteins, hormones, metabolic rate, and even disease prognosis. It also acts as a zeitgeber, or time-giver, influencing the function of molecular clocks in mammals [26]. Furthermore, changes in CBT rhythms, including in phase and/or amplitude, may have downstream effects on circadian transcriptome and associated biological pathways.

Our study has several limitations that are worth discussing. It is primarily focused on evaluating early changes of physiology, time- and tissue-specific transcriptome, and associated modifications in biological processes following exposure to IH. Proteome or metabolome analyses may also provide additional insight into downstream processes. The primary objective of this study was to assess early manifestations of disease processes by evaluating transcriptional changes, physiological parameters, and possible associated biological pathways. However, a chronic study will be required to assess long-term changes and associated pathophysiological outcomes as a means to definitively link these molecular changes to end-organ damage. We targeted 6 tissues thought to be associated with comorbid conditions among patients with OSA. Follow-up studies can expand this to other tissues to further understand the effects of IH on the whole body. Although we used a well-established mouse model replicating OSA, like any other animal models, it is possible that our IH model does not wholly represent disease seen in humans. The IH paradigm used in the current study can produce different molecular and physiological responses in different species, strains, or mice with distinct characteristics (age, gender, weight, diet). Compared to male patients, females with OSA require more time to fall asleep, have higher percentages of slow wave sleep [62], are less likely to demonstrate classic symptoms of OSA [63–65], and are more likely to have milder OSA that is REM sleep predominant [66,67]. The upstream molecular processes to explain these differences are not known. Future long-term studies should evaluate effects of IH on female mice specifically.

In summary, our findings demonstrate a unique relationship between early exposure to IH and distinct changes in biological pathways that contribute to physiological outcomes.

## Materials and methods

### Ethics statement

The mice used in this study were housed at the animal facility of Cincinnati Children’s Hospital Medical Center. Strict adherence to the Institutional Animal Care and Use Committee (IACUC) guidelines ensured the highest standards of animal welfare. Our program abides by all regulations of procurement, conditioning and quarantine, housing, management, veterinary care, and carcass disposal as set by the NIH guide for the Care and Use of Laboratory Animals. The IACUC granted approval for the animal experiments performed in this research under the protocol number IACUC 2019–0028.

### Study design

To study OSA in animals, a common approach involves exposing mice to varying levels of room air (measured as fraction of inspired oxygen [FiO_2_] approximately 21%) and different nadir values, typically ranging from 5% to 15% [68–71]. These fluctuations in FiO_2_ result in oxygen saturation (SaO_2_) nadir values of around 50% to 70% in mice, which closely corresponds to the partial pressure of oxygen (PaO_2_) values observed in humans with OSA [56,71–73]. Notably, PaO_2_ levels in humans are lower than those of mice at any given SaO_2_ value [71]. Thus the 50% to 70% SaO_2_ nadir values in mice are equivalent to 70% to 90% SaO_2_ nadir values in humans, as is commonly observed in patients with OSA. In our research, IH was achieved by reducing the 21% fractional inhaled oxygen to 8% over a period of roughly approximately 30 seconds followed by an immediate 15-second recovery period to 21% oxygen using the OxyCycler A84XOV (Biospherix, Parish, NY, USA). The fractional oxygen concentration was then maintained at 21% for several seconds before repeating the cycle, which resulted in approximately 50 hypoxic events per hour. This regimen was designed to mimic clinically severe OSA levels commonly seen in humans.

A group of C57BL/6J male mice was purchased from Jackson Laboratory (#000664) and housed in the Cincinnati Children’s Hospital Medical Center (CCHMC) animal facility under 12 hours of light and 12 hours of darkness (LD 12:12 hours) for 7 days. The mice (*n* = 4 for each condition) used for physiological measurements were taken to the operating room, and a DSI HD-X10 telemetry probe was implanted into the carotid artery of 8-week-old mice. Following the surgery, the mice were allowed to recover for 2 weeks under LD 12:12 hours. We then exposed mice to normoxic or IH conditions for 7 days during the inactive phases (light). All mice were exposed to normoxic conditions during the active phases (dark). On the seventh day, we measured the impact of IH on various physiological parameters (CBT, HR, BP, and activity with a DSI telemetry system during the active phase. Another group of 8-week-old mice (*n* = 3 at each time point for each condition) was exposed to normoxic or IH conditions for 7 days and then was released into constant normoxic darkness for 2 days. This approach aimed to assess the impact of short-term (7-day) IH exposure on circadian rhythms without any influence of diurnality (12 hours light and 12 hours dark) and immediate effects of exposure conditions. Multiple tissues (liver, lung, kidney, muscle, heart, and cerebellum) were harvested on the second day of constant darkness to assess changes in their transcriptome (**Fig 1**).

### Surgical method for implanting DSI HD-X10 telemetry

The surgical procedure was performed on 8-week-old C57BL/6J mice [74]. Prior to surgery, mice were anesthetized with pentobarbital, supplemented with isoflurane and ketamine. Fully anesthetized mice were injected subcutaneously with the analgesic buprenorphine hydrochloride (0.03 mg/ml diluted Buprenex; 0.01 to 0.05 mg/kg) to prevent pain upon awakening. Artificial tears were placed over the eyes, and then the hair at the surgical site was removed using NAIR hair remover. The surgical site was prepared aseptically by cleaning the skin 3 times with betadine and alcohol using a surgical scrub. A 1-cm incision was made on the neck to expose the submandibular area. Careful dissection was performed around the trachea and great vessels. We isolated the left carotid artery and placed 2 silk sutures beneath the vessel, one proximally and one distally along the artery. A distant suture was used to ligate the artery permanently by tying it off at the level of the mediastinum. A small incision was made on the carotid artery to secure the HD-X10 catheter in the carotid artery and then secured using the proximal and distal sutures. We then created a subcutaneous tunnel to secure the transmitter in the flank region. After surgery, sutures and tissue adhesive were used to close the skin. In addition, mice were administered 5 mg/kg of diluted 1 mg/ml Carprofen subcutaneously. Mice were kept warm throughout with circulating water. One day after surgery, mice were housed in LD 12:12 hours for a 2-week recovery.

### Analysis of physiological parameters

The mice with transmitters were housed in individual cages. We placed each cage on a receiver (RPC-1; DSI) to collect the physiological data (BP and CBT) via radiofrequency from the transmitter. Data were transmitted from the receivers to the Matrix 2.0 (MX2; DSI), which managed communication between the receivers and the data acquisition computer. Furthermore, the Ambient Pressure Reference Monitor (APR-2; DSI) provided dynamic corrections by measuring atmospheric pressure and transmitting a digital signal to the computer. Data were acquired every 5 minutes using the DSI Ponemah data acquisition system and analyzed with the Ponemah Software v6.51. Due to technical limitations with recording telemetry data during hypoxic exposure, measurements were only taken during the 12-hour active phase of the mice. To assess both time-independent overall effects and time-dependent 1-hour bin-based effects, we used a two-way ANOVA for statistical analysis, with statistical significance set at *p* ≤ 0.05.

### RNA extraction and sequencing

The RNA from all tissues was extracted using a Trizol method. All frozen tissues were homogenized in liquid nitrogen with a mortar and pestle. Following that, we mixed 1000 μl of ice-cold TRIzol (Invitrogen, #15596018, Carlsbad, CA, USA) with 100 mg of ground tissue and added 200 μl of chloroform (Fisher Chemical, #C298-500, Fair Lawn, NJ, USA). After mixing, the sample was incubated at room temperature for 3 minutes and centrifuged at 12,000 × *g* for 15 minutes at 4°C. The top aqueous phase was collected into a new Eppendorf tube and an equal amount of isopropanol (Sigma Aldrich, #437522-4L, Saint Louis, MO, USA) was added. After mixing well and incubating at room temperature for 10 minutes, the mixture was centrifuged at 12,000 × *g* for 10 minutes at 4°C. The supernatant was discarded, and the RNA pellet was washed 3 times with ice-cold 70% ethanol (Koptec, #V1016, King of Prussia, PA, USA). After washing, the pellet was air-dried for 5 to 10 minutes and dissolved in 25 μl of UltraPure DNase/RNase-Free Distilled Water (Invitrogen, #10977015, Carlsbad, CA, USA).

The RNA-Seq was performed at the Genomics Center at Rutgers New Jersey Medical School. Fluorometric quantification of RNA input was performed using a Qubit 2.0 instrument (Thermo Fisher Scientific, #Q10211, Waltham, MA, USA). RNA integrity number equivalent (RINe), a measuring unit produced by Agilent 2200, was used to estimate the integrity of RNA before proceeding with library preparation (Agilent Technologies, #5067–5576, Santa Clara, CA, USA). We used only samples with RINe values of 8 or higher. For library preparation, a poly (A) selection kit, the NEBNext Ultra II Directional RNA Library Prep Kit for Illumina (New England Biolabs, #E7765L, Ipswich, MA, USA) was used to enrich the mRNA with 1 μg of total RNA according to the manufacturer’s protocol. Libraries were quantified by a Qubit instrument (Thermo Fisher Scientific, #Q33231, Waltham, MA, USA), while library quality was checked by a TapeStation 2200 with D1000 tape (Agilent Technologies, #5067–5582, Santa Clara, CA, USA). We adjusted library concentrations to 2 nM for multiplex sequencing. A sample pooling adjustment was made based on the test run of the Miseq Nano kit (Illumina, #MS-103-1001, California, United States). All samples were sequenced with Illumina Novaseq 6000 S4 Reagent Kit v1.5 (Illumina, #20028312, California, United States), achieving a median of 60 million mapped reads per sample. Illumina’s SAV was used to check the data quality. The Illumina Bcl2fastq2 Conversion Software v2.17 was used to demultiplex the data. The quality of sequences in FASTQ format was assessed using FASTQC (v0.11.3).

### Differential rhythmicity analysis

Raw FASTQ reads of each sample were aligned and quantified using an ENSEMBL mouse reference genome, release-102, by Kallisto [75]. The normalized quantification, in transcripts per million (TPM), was used for circadian analysis. We used MetaCycle and CircaCompare to test the rhythmicity [18,19]. Genes with consistently low TPM across the time course (= 0 for all, or maximum < 1) were filtered out from the circadian analysis. A MetaCycle 2D with q ≤ 0.05 (JTK CYCLE and Lomb-Scargle) was used to determine the presence of rhythms in respective conditions. Genes that passed through the q-value threshold of MetaCycle in at least 1 condition were analyzed for differential rhythms using CircaCompare. According to the CircaCompare algorithm, *p* ≤ 0.05 was used to determine the presence of circadian rhythms. For genes that were significantly rhythmic in both normoxic and IH conditions, q ≤ 0.05 of CircaCompare was used to determine the differences in amplitude, acrophase, or MESOR. Sample resolution is an important factor in circadian analysis as it affects the accuracy of phase estimates. We based our cutoffs on recently established guidelines in the field [76]. In this study, acrophase differences were only considered if they were at least 4 hours apart. This was done to ensure accurate results based on the 3-hour sampling resolution.

### Analysis of differential mean expression over 24 hours

To test for differences between normoxia and IH, we used the estimated count from Kallisto and DESeq2 R package to compare mean 24-hour expression levels between normoxic and IH conditions. A cutoff of q ≤ 0.05 and a 1.5-fold difference were used to identify genes that were up- or down-regulated in response to IH. To examine the regulatory targets associated with differential gene expression, we used mouse regulatory target gene sets (MIR and transcription factor targets) from GSEA Molecular Signature Database v7.5.1 [15]. We also performed GSEA using hallmark gene sets from the same database to determine the most affected biological pathways by IH exposure. Statistical cutoff q ≤ 0.05 was used to identify the associated regulated targets and altered pathways across the tissues.

### Correlation analysis between differential circadian rhythms and mean 24-hour expression

To evaluate the connection between genes showing disparate circadian rhythms and the mean 24-hour expression for each tissue, we employed Fisher’s exact test. A *p* ≤ 0.05 was considered for significant correlation. We further examined the genes that displayed varied rhythmic expression and differential mean expression over 24 hours following normoxic versus IH conditions. We then performed gene enrichment analysis for mammalian phenotypes using Enrichr [20] on the overlapping genes. A statistical significance of *p* ≤ 0.01 was used to identify the potential effects of IH on mammalian phenotype.

### Bioluminescence assay

To evaluate the changes of the circadian clock at the protein level, the circadian rhythms of clock gene PER2 were assessed in the lung of PER2^*Luc*^ mice following exposure to short-term normoxia or IH. In this assay, fresh lungs were collected from mice exposed to normoxic or IH conditions and were sliced into 75-μm pieces. Four pieces of the lung from each mouse were placed into the 35-mm petri dishes with 800 μl of filter sterilized culture medium (DMEM powder with 0.35 g NaHCO3, 3.5 g D-Glucose, 10 mM HEPES, 0.025 g Pen-Strep, and 1 × B27 supplement and make up to 1 L using ddH2O). After 48 hours, 800 μl of recording medium (culture medium with final concentration of 0.1 mM Luciferin) was replaced with tissue culture medium. The Lumicycle 32 (Actimetrics) was used to measure the bioluminescence signal at 37°C in constant darkness. The data were analyzed using WAVECLOCK R package [77]. An unpaired *t* test with *p* ≤ 0.01 was used to determine the statistical significance for differences in amplitude, MESOR, and acrophase.

## Supporting information

S1 Fig Volcano plots for representing genes with differential mean 24-hour expression after exposure to normoxia vs. IH. **(A)** Liver, **(B)** lung, **(C)** kidney, **(D)** muscle, **(E)** heart, and **(F)** cerebellum. The significant differences for up- (Orange) or down (blue)-regulated genes were determined using the statistical cutoff q ≤ 0.05 of DESeq2 with 1.5-fold difference. Data output associated with S1 Fig can be found in S1 Data. Raw and processed data files accessible through GEO series accession number GSE214530.
(TIF)

S2 Fig Tissue-specific gene expression of PHDs and HIFs after exposure to normoxia and IH. **(A)** Liver, **(B)** lung, **(C)** kidney, **(D)** muscle, **(E)** heart, and **(F)** cerebellum. The expression of *Egln2*, *Arnt*, and *Hif3a* in the lung, *Egln3* in the heart, and *Egln2* in the cerebellum was found to be significantly altered by IH (*p* ≤ 0.05, two-way ANOVA*)*. Data for S2 Fig are accessible through GEO series accession number GSE214530. HIF, hypoxia-inducible factor; IH, intermittent hypoxia; PHD, prolyl hydroxylase; TPM, transcripts per million.
(TIF)

S3 Fig Heatmaps for genes with differential rhythms after exposure to normoxia vs. IH. A heatmap shows genes with 3 categories, including loss or gain of rhythm after exposure to IH as well as rhythmic in both normoxic and IH conditions for **(A)** liver, **(B)** kidney, **(C)** muscle, and **(D)** heart. **(E)** The cerebellum contains only 2 rhythmic genes. Therefore, it is not represented in the heatmap. The composition of rhythmic genes in each category varied by tissue. MetaCycle (q ≤ 0.05) and CircaCompare (*p* ≤ 0.05) were used to determine the significance. In these tissues, only the canonical clock genes in the heart and muscle were significantly impacted by IH. **(F)** Both *Cry1* and *Rorc* gained rhythms in the heart after IH exposure. **(G)** *Cry2*, *Hlf*, *Bhlhe41*, and *Nfil3* showed MESOR changes in the heart. (**H**) *Hlf* showed MESOR change in the muscle. Data output of S3 Fig can be found in S4 Data. Raw and processed data files for S3 Fig are accessible through GEO series accession number GSE214530. IH, intermittent hypoxia; MESOR, Midline Estimating Statistics of Rhythm; TPM, transcripts per million.
(TIF)

S4 Fig Up-regulated genes with differential circadian rhythms in the lung following IH exposure. A significant **(A)** loss or **(B)** gain of rhythms, **(C)** differences in amplitude and MESOR, and **(D)** differences with MESOR after exposure to IH. MetaCycle (q ≤ 0.05) and CircaCompare (*p* ≤ 0.05) were used to determine the presence of circadian rhythms. For genes that exhibited significant rhythmicity under both normoxic and IH conditions, q ≤ 0.05 of CircaCompare was used to determine the differences in amplitude, acrophase, or MESOR. Statistical cutoff q ≤ 0.05 of DESeq2 with 1.5-fold difference was used to determine the up-regulated genes. Data output of S4 Fig is associated with S1 Data and S4 Data. Raw and processed data files for S4 Fig are accessible through GEO series accession number GSE214530. IH, intermittent hypoxia; MESOR, Midline Estimating Statistics of Rhythm; TPM, transcripts per million.
(TIF)

S5 Fig Down-regulated genes with differential circadian rhythms in the lung following IH exposure. A significant **(A)** loss or **(B)** gain of rhythms and **(C)** differences with MESOR after exposure to IH. MetaCycle (q ≤ 0.05) and CircaCompare (*p* ≤ 0.05) were used to determine the presence of circadian rhythms. For genes that exhibited significant rhythmicity under both normoxic and IH conditions, q ≤ 0.05 of CircaCompare was used to determine the differences in amplitude, acrophase, and MESOR. Statistical cutoff q ≤ 0.05 of DESeq2 with 1.5-fold difference was used to determine the down-regulated genes. Data output of S5 Fig is associated with S1 and S4 Data files. Raw and processed data files for S5 Fig are accessible through GEO series accession number GSE214530. IH, intermittent hypoxia; MESOR, Midline Estimating Statistics of Rhythm; TPM, transcripts per million.
(TIF)

S6 Fig Up- and down-regulated genes with differential circadian rhythms in the liver and the heart upon IH exposure. Up-regulated genes with **(A)** loss of rhythms and **(B)** differences with MESOR following exposure to IH in the liver. Down-regulated genes with a significant **(C)** loss of rhythms or **(D)** differences with MESOR after IH exposure in the liver. **(E)** Up-regulated genes with MESOR differences in the heart. **(F)** Down-regulated genes with gain of rhythms in the heart. MetaCycle (q ≤ 0.05) and CircaCompare (*p* ≤ 0.05) were used to determine the presence of circadian rhythms. For genes that exhibited significant rhythmicity under both normoxic and IH conditions, q ≤ 0.05 of CircaCompare was used to determine the differences in amplitude, acrophase, or MESOR. Statistical cutoff q ≤ 0.05 of DESeq2 with 1.5-fold difference was used to determine the up- or down-regulated genes. Data output of S6 Fig is associated with S1 and S4 Data files. Raw and processed data files for S6 Fig are accessible through GEO series accession number GSE214530. IH, intermittent hypoxia; MESOR, Midline Estimating Statistics of Rhythm; TPM, transcripts per million.
(TIF)

S1 Data Tissue-specific differential mean 24-hour expression analysis. (XLSX)

S2 Data Tissue-specific enrichment analysis for regulatory targets. (XLSX)

S3 Data Tissue-specific enrichment analysis for hallmark pathways. (XLSX)

S4 Data Tissue-specific differential circadian rhythm analysis. (XLSX)

S5 Data Bioluminescence data of the lung PER2 ^*LUC*^. (XLSX)

S6 Data Mammalian phenotype enrichment analysis. (XLSX)

S7 Data Real-time data of physiological parameters. (XLSX)

## Abbreviations

BP: blood pressure
CBT: core body temperature
EMT: epithelial mesenchymal transition
ERE: estrogen response early
GSEA: gene set enrichment analysis
HIF: hypoxia-inducible factor
HR: heart rate
IH: intermittent hypoxia
MESOR: Midline Estimating Statistics of Rhythm
MIR: microRNA
OSA: obstructive sleep apnea
PHD: prolyl hydroxylase
TPM: transcripts per million
