# Supplementary figures and images for: Obstructive sleep apnea in a mouse model is associated with tissue-specific transcriptomic changes in circadian rhythmicity and mean 24-hour gene expression
Source: PLoS Biol. 2023 May 30;21(5):e3002139. doi: 10.1371/journal.pbio.3002139 (PMC10228805; doi:10.1371/journal.pbio.3002139)

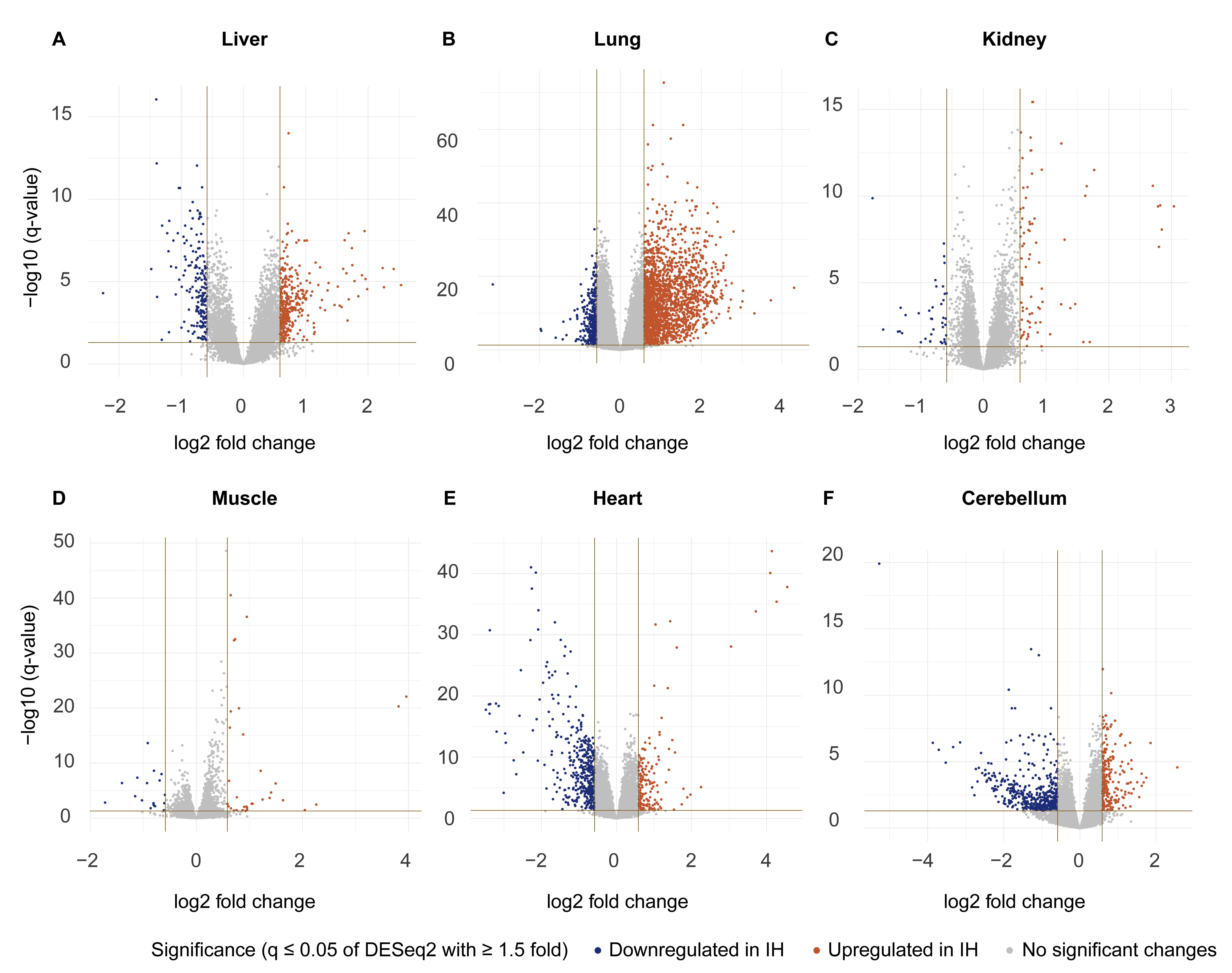

Supplement: S1 Fig — (A) Liver, (B) lung, (C) kidney, (D) muscle, (E) heart, and (F) cerebellum. The significant differences for up- (Orange) or down (blue)-regulated genes were determined using the statistical cutoff q ≤ 0.05 of DESeq2 with 1.5-fold difference. Data output associated with S1 Fig can be found in S1 Data. Raw and processed data files accessible through GEO series accession number GSE214530. (TIF) [file pbio.3002139.s001.tif]
